# TRAINING THE NEW SCIENTISTS OF AGING: BUILDING ON GEROSCIENCE OPPORTUNITIES

**DOI:** 10.1093/geroni/igad104.0940

**Published:** 2023-12-21

**Authors:** LaDora Thompson

**Affiliations:** Boston University, Boston, Massachusetts, United States

## Abstract

Geroscience is focused on improving health and well-being by beneficially altering or reversing the cellular and molecular changes that take place as we age. These changes, uncovered by the biology of aging research, can contribute to a progressive loss of function and the onset of frailty and many diseases prevalent at older ages. In the 21st century there is a need for increased engagement with researchers and clinicians across multiple disciplines to translate these geroscience discoveries more effectively into new medical advances. This presentation will highlight efforts to increase awareness and understanding of these geroscience discoveries to stakeholder groups and to build a more diverse and interdisciplinary geroscience workforce, including researchers, clinicians, and members of the public. To date, there are creative education programs aimed at enhancing and expanding broader awareness of geroscience research (e.g., skills development, hands-on research experiences, formal curriculum informed in the science of aging, engagement activities dealing with ethical, economic, political and societal ramifications of extending human lifespan, development of shared methods, networking with experts in the geroscience field, materials and outreach programs on the topic of geroscience), which target basic, translational, clinical researchers and members of the public. Lastly, this presentation will outline the activities within the Translational Rehabilitation in Geroscience Initiative and the Translational Geroscience Network, a collaboration of researchers looking at clinical interventions that target fundamental mechanism of aging to delay, prevent or treat age-related diseases and disabilities as a group, instead of one at a time, enabling more-rapid translation of therapies.

